# Risk factors and in-hospital mortality of postoperative hyperlactatemia in patients after acute type A aortic dissection surgery

**DOI:** 10.1186/s12872-021-02244-7

**Published:** 2021-09-11

**Authors:** Su Wang, Dashuai Wang, Xiaofan Huang, Hongfei Wang, Sheng Le, Jinnong Zhang, Xinling Du

**Affiliations:** 1 Department of Emergency Medicine, Union Hospital, Tongji Medical College, Huazhong University of Science and Technology, Wuhan, 430022 China; 2 Department of Cardiovascular Surgery, Union Hospital, Tongji Medical College, Huazhong University of Science and Technology, Wuhan, 430022 China

**Keywords:** Postoperative hyperlactatemia, Stanford type A acute aortic dissection, Risk factor, Prediction model, Nomogram

## Abstract

**Background:**

Hyperlactatemia may be caused by increased production due to tissue hypoxia or non-hypoxia. The aim of this study was first to identify risk factors for postoperative hyperlactatemia (POHL) after Stanford type A acute aortic dissection surgery (AADS) and construct a predictive model, and second to evaluate the impact of POHL on prognosis.

**Methods:**

This retrospective study involved patients undergoing AADS from January 2016 to December 2019 in Wuhan Union Hospital. Multivariate logistic regression analysis was performed to identify independent risk factors for POHL. A nomogram predicting POHL was established based on these factors and was validated in the original dataset. The receiver operating characteristic curve was drawn to assess the ability of postoperative lactate levels to predict the in-hospital mortality.

**Results:**

A total of 188 patients developed POHL after AADS (38.6%). Male gender, surgery history, red blood cell transfusion and cardiopulmonary bypass time were identified as independent predictors. The C-index of the prediction model for POHL was 0.72, indicating reasonable discrimination. The model was well calibrated by visual inspection and goodness-of-fit test (Hosmer–Lemeshow χ^2^ = 10.25, *P* = 0.25). Decision and clinical impact curves of the model showed good clinical utility. The overall in-hospital mortality rate was 10.1%. Postoperative lactate levels showed a moderate predictive power for postoperative in-hospital mortality (C-index: 0.72).

**Conclusion:**

We developed and validated a prediction model for POHL in patients undergoing AADS, which may have clinical utility in personal risk evaluation and preventive interventions. The POHL could be a good predictor for in-hospital mortality.

**Supplementary Information:**

The online version contains supplementary material available at 10.1186/s12872-021-02244-7.

## Introduction

Lactate levels have been used as a marker of illness severity and measurements in response to therapeutic interventions. Elevated lactate has been found to be associated with increased mortality in many diseases such as trauma, sepsis and cardiac arrest [1]. Postoperative hyperlactatemia (POHL) is frequently observed in patients undergoing cardiac surgery, which is also related to postoperative adverse events [2–4]. As a catastrophic disease, although emergency surgery is required to perform in most cases, Stanford type A acute aortic dissection still maintains a high early mortality, up to 15.1% [5, 6].

Preoperative hyperlactatemia was recognized to predict in-hospital mortality in patients with Stanford type A acute aortic dissection in some studies [7, 8]. Nevertheless, some other studies suggested that both preoperative hyperlactatemia and POHL had poor predictive performance for mortality [9]. To our knowledge, researches on the effect of POHL on the outcomes of patients undergoing Stanford type A acute aortic dissection surgery (AADS) are currently limited, and the association between POHL and mortality remains to be clarified.

The therapy aimed at maintaining appropriate lactate concentrations after cardiac surgery could decrease morbidity and shorten hospital stay, but there are still challenges in reducing costs and improving the use of resources for cardiac surgery and anesthesia [10]. Cardiopulmonary bypass (CPB) flow rate, CPB duration and mean arterial blood pressure during CPB have been identified as independent predictors for POHL in patients undergoing cardiac surgery [3, 11]. However, for those with a higher disease severity, it seems particularly important to find more evidence on risk factors for POHL. Unfortunately, there are no prior studies identifying risk factors for POHL targeting patients undergoing AADS, let alone a predictive model.

The first purpose of this study was to identify independent predictors for POHL in patients undergoing AADS and to establish and validate a risk prediction model. The second purpose was to analyze the relationship between POHL and the outcomes, and to evaluate the performance of lactate levels in predicting mortality.

## Methods

### Study design and settings

This study was designed as a retrospective observational study. We screened consecutive adult patients who underwent AADS from January 2016 to December 2019 at our institution. The patients who died intraoperatively and had incomplete medical records were excluded.

### Data collection and definition

Clinical data for analysis were obtained from electronic medical record system of our institution. The selection of variables was based on previous literature and clinical practice. Preoperative variables included gender, age, body mass index, left ventricular ejection fraction, the history of smoking, drinking, surgery, hypertension, cerebrovascular disease, pericardial effusion, diabetes mellitus, chronic obstructive pulmonary disease, atrial fibrillation, pulmonary artery hypertension, gastrointestinal tract disease, peripheral vascular disease, and renal insufficiency. Laboratory values included white blood cell count, red blood cell (RBC) count, hemoglobin, platelet count, serum creatinine, albumin, and globulin. Operative variables included surgical types, aortic cross clamp time, cardiopulmonary bypass time, and blood transfusion. Postoperative variables included PH value, postoperative pneumonia, readmission to intensive care unit (ICU), reintubation, tracheotomy, in-hospital mortality, the lengths of mechanical ventilation, ICU and hospital stay.

In this study, the peak arterial blood lactate value in the first 12 h after ICU admission was recorded, and hyperlactatemia was defined as lactate level > 4 mmol/L based on previous studies [1].

### Statistical analysis

Statistical analysis was performed using SPSS (IBM SPSS Statistics 26.0, SPSS Inc., Chicago, IL) and R software (version 4.0.3, www.R-project.org/). The R packages we used included readr, rms, regplot, foreign and rmda. The used code to generate nomogram and the related internal validation plots was presented in Additional file 1. Two-tailed *P* value < 0.05 was considered statistically significant in the multivariate analysis.

Univariate analysis was first performed to screen possible risk factors for POHL after AADS. Continuous variable was expressed as mean (standard deviation) or median (inter-quartile range), and Student’s t-test or Mann–Whitney U test was applied when appropriate. Categorical variable was expressed as frequency (percentage), and chi-square or Fisher’s exact test was applied when appropriate. Variables with *P* value < 0.1 in the univariate analysis were further analyzed in the multivariate logistic regression to identify independent risk factors. Based on the multivariate model, a nomogram was established for better application.

We conducted internal validation by bootstrapping using 1000 replications. C-index or the area under the receiver operating characteristic (ROC) curve (AUC) was applied to model discrimination evaluation. Calibration curve and Hosmer–Lemeshow goodness-of-fit test were applied to model calibration assessment. Decision curve analysis was used to assess the clinical utility. The decision curve showed threshold probability against standardized net benefit. The clinical impact curve displayed the number of the judged high risk and the true positives among 1,000 patients with different threshold probability. The ability of the peak lactate concentration to predict in-hospital mortality was evaluated by calculating the AUC.

## Results

### Demographic characteristics

**Fig. 1:**
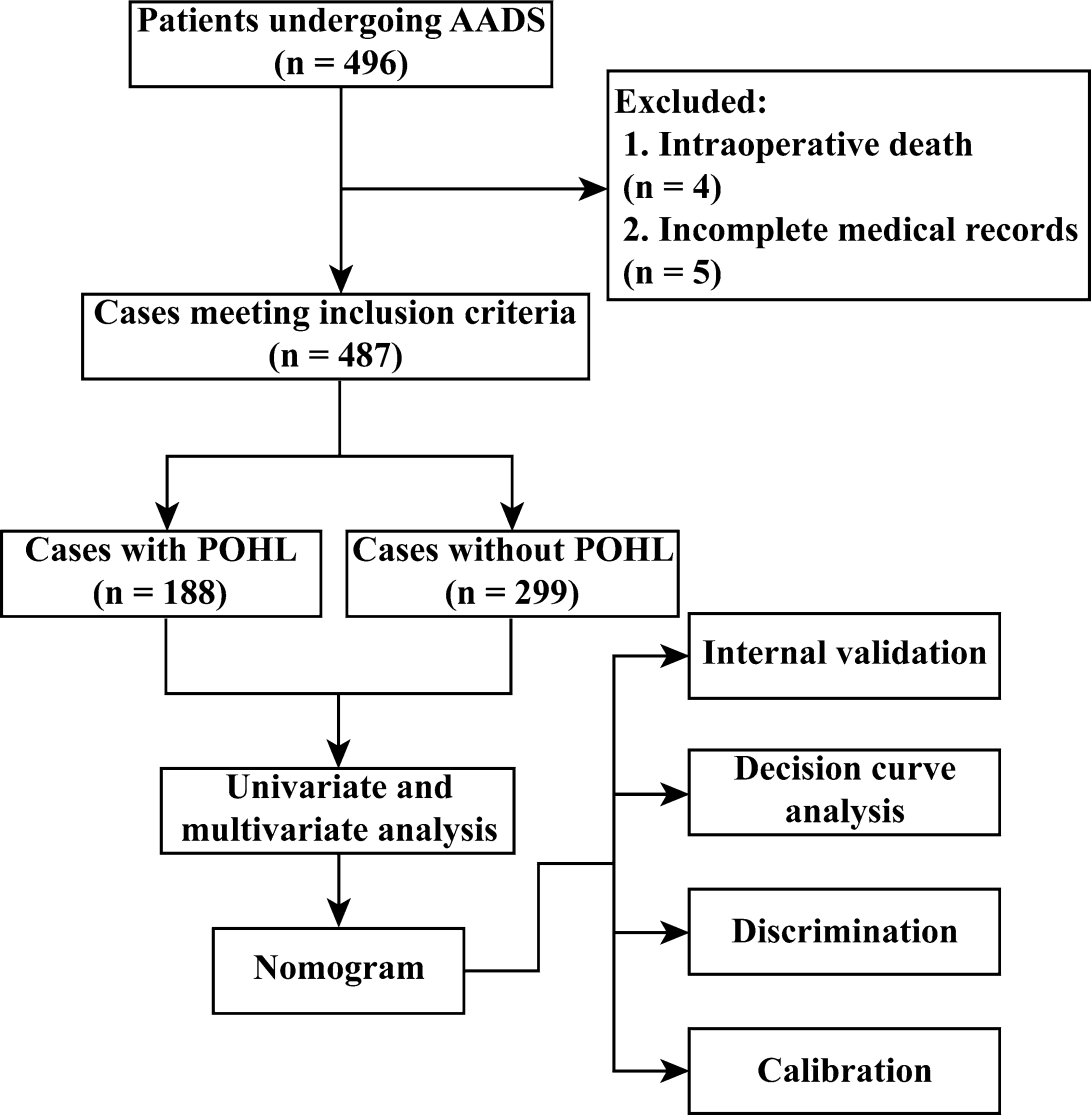
Flow chart of the study. AADS, Stanford type A acute aortic dissection surgery; POHL, postoperative hyperlactatemia

Among the 496 patients who underwent AADS, four died intraoperatively and five had incomplete medical records (Fig. 1). The remaining 487 patients were included for further analysis. The average age of the patients was 49.68 ± 11.35 years, and more than three-quarters were male. The overall incidence of POHL after AADS reached 38.6%.

Of the 487 operations, combined valve surgery was performed for 110 cases (22.6%), combined coronary artery bypass grafting for 25 cases (5.1%), combined valve and coronary surgery for 26 cases (5.3%), and combined other types of cardiac surgery for 7 cases (1.4%).

The common comorbidities among patients were smoking history (43.7%), drinking history (35.7%), hypertension (68.2%), pericardial effusion (26.7%), and surgery history (24.8%). The median CPB duration was 211 (175, 256) minutes and the aortic cross clamp duration was 120 (97, 147) minutes. The median volume of the intraoperative RBC transfusion was 5 (4, 7) units.

### Development of the nomogram model

**Fig. 2:**
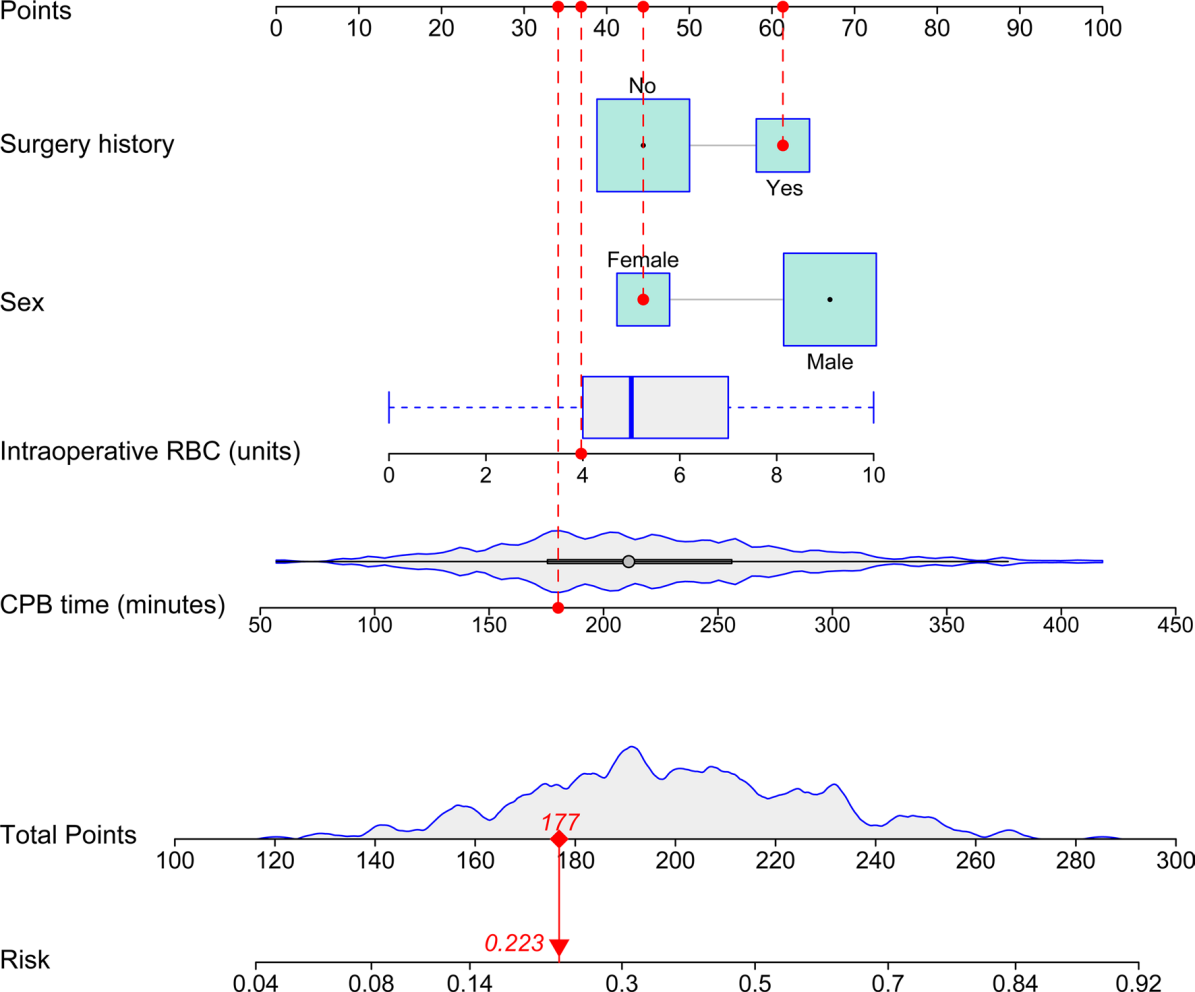
Nomogram for the prediction of postoperative hyperlactatemia. Given values of the four variables, the patient can be mapped onto the nomogram. Each red dot represents the value of each variable of the patient. The total point is 177, corresponding to a probability of 22.3% for developing postoperative hyperlactatemia. CPB, cardiopulmonary bypass; RBC, red blood cell

The comparison between patients with and without POHL are presented in Table 1. Before the building of a multivariate model, collinearity diagnostic tests were performed. Factors with *P* value < 0.1 in the univariate analysis were further analyzed in the multivariate analysis and four independent predictors for POHL after AADS were identified, including male gender, surgery history, intraoperative transfusion of RBC and CPB time (Table 2). Based on the multivariate model, a nomogram used to predict the risk of POHL was then constructed (Fig. 2). Regression coefficients of the variables were scaled to points within the range of 0—100, reflecting their relative importance.

**Table 1:** Univariate analysis of possible risk factors for POHL after AADS

Characteristic	Without POHL n = 299 (%)	With POHL n = 188 (%)	*P* value
*Demographics*			
Male	216 (72.2)	152 (80.9)	0.031
Age (years)	49.39 ± 11.63	50.14 ± 10.92	0.477
Body mass index (kg/m^2^)	25.15 ± 3.80	25.60 ± 3.49	0.182
Smoking history	124 (41.5)	89 (47.3)	0.204
Drinking history	104 (34.8)	70 (37.2)	0.583
*Underlying conditions*			
Hypertension	199 (66.6)	133 (70.7)	0.334
Diabetes mellitus	16 (5.4)	5 (2.7)	0.155
Chronic obstructive pulmonary disease	3 (1.0)	2 (1.1)	0.949
Cerebrovascular disease	51 (17.1)	37 (19.7)	0.464
Peripheral vascular disease	40 (13.4)	25 (13.3)	0.980
Renal insufficiency	91 (30.4)	78 (41.5)	0.013
Gastrointestinal tract disease	22 (7.4)	20 (10.6)	0.209
Atrial fibrillation	4 (1.3)	0 (0)	0.304
Surgery history	71 (23.7)	50 (26.6)	0.479
Pulmonary artery hypertension	10 (3.3)	4 (2.1)	0.581
Pericardial effusion	88 (29.4)	42 (22.3)	0.085
Left ventricular ejection fraction (%)	62 (60, 65)	62 (60, 65)	0.945
*Laboratory values*			
White blood cell count (× 10^9^/L)	9.8 (7.5, 12.5)	10.4 (7.8, 13.3)	0.228
Red blood cell count (× 10^12^/L)	4.2 (3.7, 4.5)	4.3 (3.9, 4.6)	0.030
Hemoglobin (g/l)	125 (111, 138)	131 (119, 141)	0.005
Platelet count (× 10^9^/L)	162 (126, 210)	154 (126, 186)	0.120
Serum creatinine (μmol/L)	78.0 (65.2, 109.0)	83.0 (69.0, 115.5)	0.164
Serum albumin (g/L)	37.4 (34.4, 40.7)	38.3 (35.7, 41.0)	0.048
Serum globulin (g/L)	25.7 (22.6, 28.5)	25.3 (23.0, 28.0)	0.625
*Operative variables*			
Surgical types			0.003
Isolated AADS	209 (69.9)	110 (58.5)	
Combined valve surgery	68 (22.7)	42 (22.3)	
Combined coronary artery bypass grafting	9 (3.0)	16 (8.5)	
Combined valve and coronary surgery	10 (3.3)	16 (8.5)	
Combined other types of cardiac surgery	3 (1.0)	4 (2.1)	
Cardiopulmonary bypass time (minutes)	199 (162, 239)	233 (197, 274)	< 0.001
Aortic cross clamp time (minutes)	116 (94, 142)	130 (104, 155)	< 0.001
Transfusion of red blood cells (units)	4 (3, 6)	6 (4, 8)	< 0.001

AADS, Stanford type A acute aortic dissection surgery; POHL, postoperative hyperlactatemia

**Table 2:** Multivariate analysis of independent risk factors for POHL after AADS

Characteristic	Coefficient	Standard error	OR (95% CI)	*P* value
Male	0.720	0.248	2.055 (1.264–3.339)	0.004
Surgery history	0.539	0.231	1.714 (1.090–2.693)	0.020
Transfusion of RBC (units)	0.187	0.051	1.206 (1.091–1.332)	< 0.001
CPB time (minutes)	0.009	0.002	1.009 (1.005–1.012)	< 0.001
Intercept	− 4.118	0.504	0.016	< 0.001

AADS, Stanford type A acute aortic dissection surgery; CI, confidence interval; CPB, cardiopulmonary bypass; OR, odds ratio; POHL, postoperative hyperlactatemia; RBC, red blood cell

On the basis of the four predictors, a patient can be mapped to the nomogram. The red dot in each line of the nomogram represented the corresponding value. Points were translated into probabilities through logit transformation. The probability of POHL after AADS in a particular patient can be easily calculated by summing the points of all associated predictors. A male patient who had surgery history, more RBC transfusion and longer CPB time had higher points and thus higher risk of POHL after AADS. The probability of POHL predicted by this nomogram ranged from 0.04 to 0.92.

### Assessment and validation of the nomogram model

**Fig. 3:**
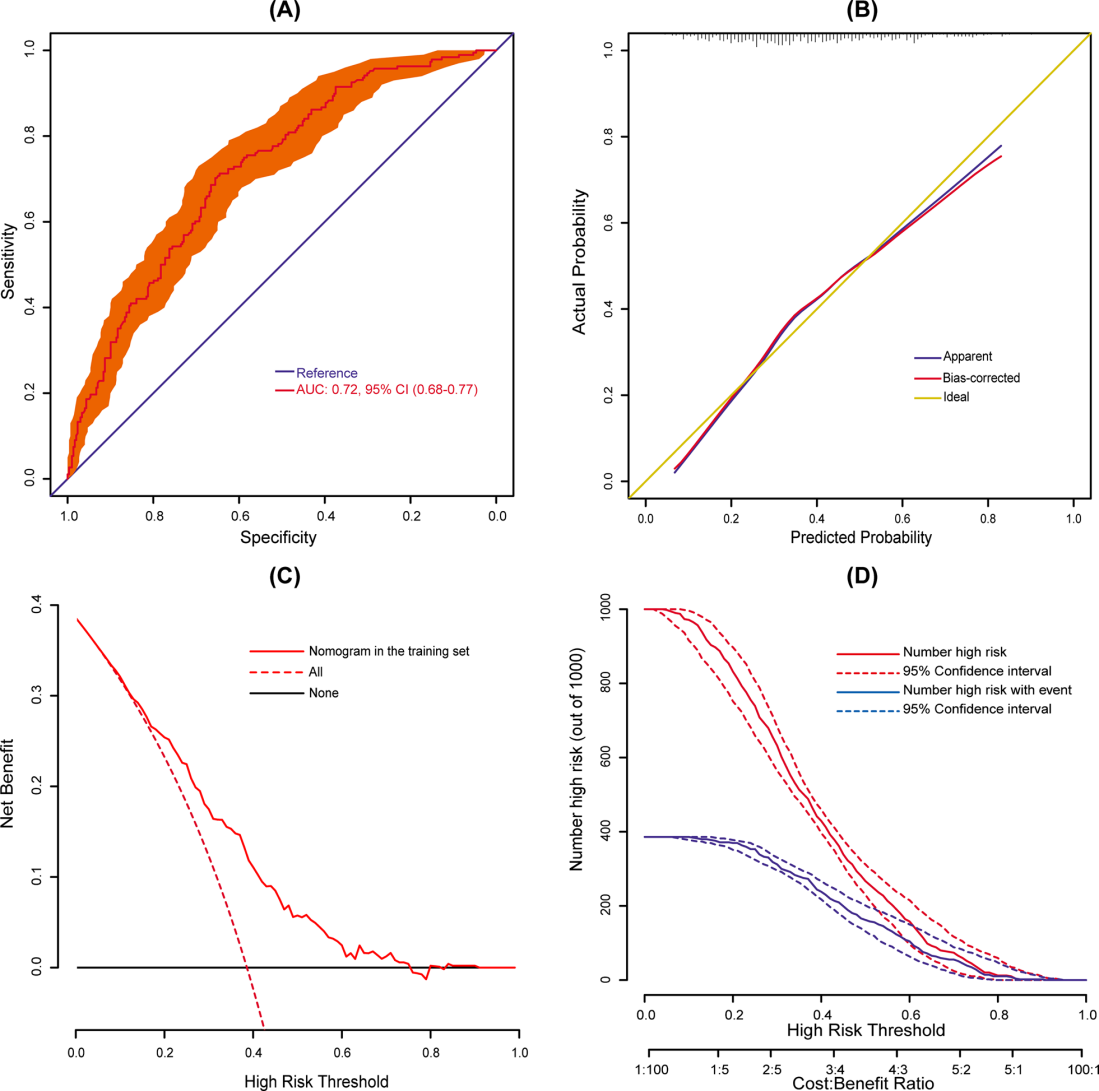
Assessment and validation of the nomogram model. **A** ROC curve for the nomogram; **B** calibration plot of the nomogram. Gold line (diagonal 45-degree broken line) represents perfect prediction that nomogram-predicted probability (x-axis) matches actually observed possibility (y-axis). Blue line indicates unadjusted calibration accuracy. Red line indicates bootstrap corrected calibration accuracy. The closer the line fit is to the gold line, the better the prediction accuracy of the nomogram; **C** decision curves of the nomogram. Black line is the net benefit of intervening no patients. Dotted red line is the net benefit of intervening all patients. Solid red line is the net benefit of intervening patients on the basis of the nomogram. If personal threshold probability ranges from approximately 15% to 75%, the nomogram model can be beneficial for making the decision to intervene; and **D** clinical impact curves of the nomogram. Two horizontal axes show the correspondence between cost:benefit ratio and risk threshold. Of 1,000 patients, solid red line shows the total number of high-risk patients for each risk threshold. Solid blue line shows how many of those are with positive event. If a 20% risk threshold is used, then of 1,000 patients with AADS, about 850 are high risk, with about 380 of these developing the POHL. ROC, receiver operating characteristic; AUC, area under the receiver operating characteristic curve; CI, confidence interval; AADS, Stanford type A acute aortic dissection surgery; POHL, postoperative hyperlactatemia

Internal validation was performed using bootstrap with 1000 replicates. The ROC curve was drawn to assess the predictive capability of the nomogram (Fig. 3A). The AUC was 0.72 (95% CI: 0.68–0.77), indicating reasonable discrimination. The Hosmer–Lemeshow goodness-of-fit test was performed, indicating good calibration (Hosmer–Lemeshow χ^2^ = 10.25, *P* = 0.25). The calibration curve was plotted in Fig. 3B, which also showed good calibration by visual inspection. The decision curve analysis indicated that compared with “no intervention” or “intervention for all” strategy, using the nomogram model could get more clinical net benefits (Fig. 3C). The clinical impact curve also exhibited good predictive power and clinical utility (Fig. 3D).

### Outcome

**Fig. 4:**
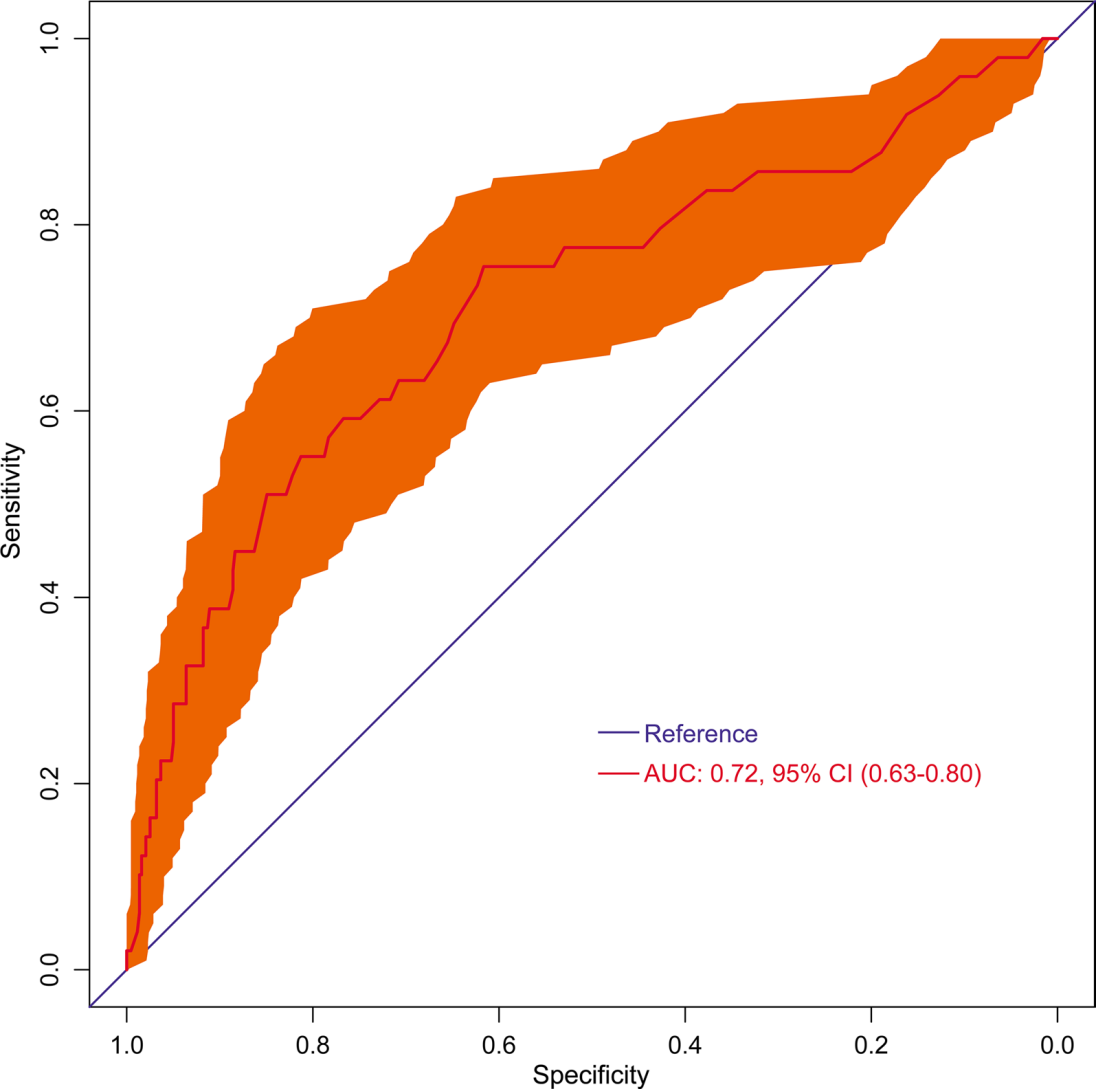
Receiver operating characteristic curve assessing the predictive ability of postoperative lactate levels with in-hospital mortality. AUC, area under the receiver operating characteristic curve; CI, confidence interval

The overall mortality rate was 10.1%, with a rate of 5.0% in patients without POHL versus 18.1% in those with POHL (*P* < 0.001). The average lactate concentration was respectively 3.92 ± 2.90 mmol/L in survivors and 7.06 ± 4.64 mmol/L in non-survivors (*P* < 0.001). The predictive power of postoperative lactate concentrations for mortality was moderate, with an AUC of 0.72 (95% CI: 0.63–0.80) (Fig. 4).

In addition, the incidence rates of postoperative acidosis, postoperative pneumonia and tracheotomy were significantly higher in POHL group compared with non-POHL group. Meanwhile, extended mechanical ventilation time, ICU stay and hospital stay were also observed in POHL group. Details of comparison between two groups are presented in Table 3.

**Table 3:** Postoperative variables in patients with and without POHL after AADS

Variables	All patients n = 487 (%)	Without POHL n = 299 (%)	With POHL n = 188 (%)	*P* value
PH < 7.35	72 (14.8)	9 (3.0)	63 (33.5)	< 0.001
Postoperative pneumonia	167 (34.3)	86 (28.8)	81 (43.1)	0.001
Reintubation	70 (14.4)	36 (12.0)	34 (18.1)	0.064
Tracheotomy	54 (11.1)	23 (7.7)	31 (16.5)	0.003
Mechanical ventilation (hours)	65 (40, 138)	59 (38, 90)	88 (46, 206)	< 0.001
Readmission to ICU	43 (8.8)	28 (9.4)	15 (8.0)	0.600
ICU stay (days)	7 (5, 11)	6 (4, 9)	8 (6, 14)	< 0.001
Hospital stay (days)	21 (17, 27)	21 (16, 27)	22 (18, 28)	0.049
Mortality	49 (10.1)	15 (5.0)	34 (18.1)	< 0.001

AADS, Stanford type A acute aortic dissection surgery; ICU, intensive care unit; POHL, postoperative hyperlactatemia

## Discussion

This study indicated the following: (1) Male gender, surgery history, more transfusion of RBC and longer CPB time were independent predictors for POHL after AADS. (2) The nomogram model we built performed well in discrimination, calibration, and clinical utility. (3) Postoperative lactate level was a good predictor for in-hospital mortality in patients undergoing AADS, and POHL was associated with poor outcomes.

Hyperlactatemia and lactic acidosis are common in patients with septic shock or severe sepsis [12]. According to our findings, these two were confirmed to occur frequently in patients undergoing AADS. The observed incidence of POHL in this study was 38.6%, which was higher than previously reported range in cardiac surgery [4, 13]. As expected, patients with POHL experienced longer duration of mechanical ventilation and ICU stay and were more likely to suffer from tracheotomy, in line with previous studies [3, 4]. Moreover, mortality rate among patients with POHL was much higher than those without POHL after AADS in our study. Similarly, the risk of mortality increased with postoperative lactate values [14], emphasizing the need of early identification of high-risk POHL populations.

Longer CPB time was independently correlated to the occurrence of POHL in this study, and this association was consistent with some previous study results in patients undergoing cardiac surgery [3, 15, 16]. Worrell et al. indicated that the CPB use was associated with higher levels of peak ICU lactate concentration when CPB time was longer than 2.5 h [17]. Hyperlactatemia was divided into two categories: “type A” caused by circulatory insufficiency (tissue hypoxia), and “type B” containing all other types [18]. As is known, microcirculation affects the intact oxygen delivery to the cells. Some impairment was found to exist in microcirculation during cardiac surgery, suggesting the impaired cellular oxygenation [19]. This study interpreted that CPB procedure altered microvascular perfusion, and anesthesia partially contributed to the alterations. Greenwood et al. further demonstrated the association between impaired microcirculation and POHL in cardiac surgery with CPB [20], and the inflammatory responses played a role in POHL development [21]. Mini-CPB could lead to fewer alterations of microperfusion, and its use may clinically improve outcomes in patients with high-risk surgery and in those with longer procedures [22].

Compared with some other studies conducted in cardiac surgery [4, 23], the duration of CPB in this study was relatively longer. The CPB duration in the majority of patients was 3–4 h, and the possibility of POHL increased a lot due to the very long CPB time. However, it was similar to the results of AADS-related studies at home and abroad [24, 25]. This may be attributed to the complexity and difficulty of the AADS, which implies a possibility of higher postoperative lactate levels and increased incidence of POHL than other cardiac operations and thus higher mortality.

Similar to the long CPB duration, the amount of RBC transfusion in patients undergoing AADS was also large. The majority of the patients were transfused with 4–7 units of RBC in this study, which was obviously a larger transfusion volume than other types of cardiac surgery. Even so, the results showed that the more RBCs were transfused, the higher the risk of POHL.

Microcirculatory dysfunction and mitochondrial defect that occurred in sepsis caused impaired tissue oxygen extraction [26, 27]. Inoue et al. found that patients with POHL after cardiac surgery also had reduced oxygen extraction rates during CPB [28]. However, increasing cardiac output or increasing hemoglobin was considered to be ineffective for correcting impaired tissue oxygen utilization [12]. The stored RBC undergoing storage lesion or hemolysis may accelerate organ tissue dysfunction [29, 30]. In other words, more RBC transfusion may not change the tissue hypoxia in certain areas, but worsen it and lead to more lactate production. Undeniably, blood transfusion is inevitable when more severe bleeding occurs in surgical repair for aortic dissection due to coagulation system disorder and the operation itself [31]. However, the strategy of using autologous platelet rich plasma may help reduce blood transfusion and decrease morbidity [32].

Acute type A aortic dissection affected men more commonly, and about two-thirds occurred in men [24, 33]. Male gender was found to independently predict POHL in this study. However, the correlation between the gender and POHL has been rarely described before. Evans et al. indicated that male gender was an independent risk factor for lactate clearance failure after mitral valve surgery [34]. Compared to females, it was reported that male patients who underwent AADS had significantly longer CPB and aortic cross clamp time, but required less intraoperative blood transfusion [24]. Despite greater hemodilution during CPB, women had a lower mortality risk than men, and the lower nadir hematocrit values during CPB was found to be negatively correlated with POHL [35, 36]. In our model, the exceeded score obtained as a male can be converted as an increased risk of up to 15%. The possible reason may be that men have greater stress response to the surgery, leading to increased metabolism, such as glycogenolysis, aerobic glycolysis, and endogenous catecholamines secretion [37, 38].

Our model contained four easily available variables, in which the two intraoperative factors can be controlled by the proficiency of the operation and the perfection of the technique. Although non-modifiable factors affect the ability of the clinician to prevent POHL, such as gender and surgery history, the model could be helpful to identify the patients at low risk. It is also meaningful for reducing unnecessary waste of medical resources and costs.

This study demonstrated that the postoperative lactate values had a good predictive effect on in-hospital mortality in patients undergoing AADS, whereas this finding was inconsistent with a previous study [9]. The difference may be caused by the smaller sample size and lower overall lactate levels, and the fact that only one measurement result was included in the analysis. To our knowledge, this is the first study reporting that postoperative lactate level is a significant predictor for mortality in patients who underwent AADS, in spite of only moderate predictive value. More studies focused on exploring the relationship between postoperative lactate level and postoperative death are still needed, and the effect of early lactate-guided therapy remains to be assessed [10].

Some limitations have to be noted. First, this was a single-center retrospective study, and the wide application of our model to other centers may be restricted. Second, due to the small sample size, we performed only internal validation by bootstrapping and lacked external validation. Third, patients with preoperative hyperlactatemia could not be effectively excluded due to the large amount of missing data of preoperative lactate values. Finally, we failed to collect data of some potential risk factors affecting lactate levels, such as CPB flow rate and blood glucose concentration.

## Conclusions

Our study is the first to identify risk factors and establish a prediction model for POHL in patients who underwent AADS. Male gender, surgery history, CPB time and RBC transfusion were identified as independent risk factors in multivariate analysis. The constructed nomogram showed good discrimination, calibration and clinical utility. Our model may be well used for personal risk assessment and clinical decision making in patients undergoing AADS. This study also suggested that POHL was prevalent in patients undergoing AADS, associated with poor outcomes. Postoperative lactate level was a new valuable marker for evaluating in-hospital mortality in patients undergoing AADS, and it can be easily and continuously obtained.

## Supplementary Information

**Additional file 1. Code to generate nomogram and the related internal validation plots. **

## Data Availability

The datasets used and/or analyzed during the current study are available from the corresponding author upon reasonable request.

## Abbreviations

AADS: Stanford type A acute aortic dissection surgery
AUC: Area under the receiver operating characteristic curve
CI: Confidence interval
CPB: Cardiopulmonary bypass
ICU: Intensive care unit
OR: Odds ratio
POHL: Postoperative hyperlactatemia
ROC: Receiver operating characteristic
RBC: Red blood cell
