# Supplementary material for: Risk factors and in-hospital mortality of postoperative hyperlactatemia in patients after acute type A aortic dissection surgery
Source: BMC Cardiovasc Disord. 2021 Sep 11;21:431. doi: 10.1186/s12872-021-02244-7 (PMC8436469; doi:10.1186/s12872-021-02244-7)
Supplement: Supplementary file 1 — Additional file 1. Code to generate nomogram and the related internal validation plots. [file 12872_2021_2244_MOESM1_ESM.docx]

**The generation of the nomogram and the related internal validation plots**

**First, copy the data set. Then, run the following programs.**

mydata<-read.table("clipboard",header = TRUE,sep = "\t")

mydata<-as.data.frame(mydata)

mydata$POHL<-ifelse(mydata$POHL=="1",1,0)

mydata$sex<-factor(mydata$sex,levels=c(0,1),labels=c("0","1"))

mydata$surgery history<-factor(mydata$surgery history, levels=c(0,1), labels = c("0","1"))

View(mydata)

head(mydata)

names(mydata)

summary(mydata)

str(mydata)

library(readr)

library(rms)

library(regplot)

attach(mydata)

dd<-datadist(mydata)

options(datadist='dd')

fit<-lrm(POHL~.,data=mydata)

fit

nom<-regplot(fit,observation=mydata[1,],center=T,title="Nomogram",points=T, odds=F, showP=T, rank="sd", clickable=T)

cal<-calibrate(fit,method = 'boot',B=1000)

plot(cal,xlim = c(0,1.0),ylim = c(0,1.0))
